# Suppression of weed and insect populations by living and straw mulches in sesame (*Sesamum indicum* L.)

**DOI:** 10.1038/s41598-023-48978-6

**Published:** 2023-12-07

**Authors:** Solmaz Azimi, Rouhollah Amini, Majid Hosseingolizadeh

**Affiliations:** 1 Department of Plant Protection, Faculty of Agriculture, Azarbaijan Shahid Madani University, Tabriz, Iran; 2 Department of Plant Ecophysiology, Faculty of Agriculture, University of Tabriz, Tabriz, Iran

**Keywords:** Plant ecology, Agroecology

## Abstract

In order to evaluate the effect of different weed management treatments on weeds, pest and natural enemies populations in sesame (*Sesamum indicum* L.), a 2-year study was conducted in East Azarbaijan, Iran in 2020–2021. The study was conducted based on randomized complete block design with four replications. The weed management treatments consisted of trifluralin use (960 g ai ha^−1^), wheat straw mulch (WSM), living mulches of fenugreek (*Trigonella foenum-graecum* L.) (FLM), bitter vetch (*Vicia ervilia* L.) (VLM), calendula (*Calendula officinalis* L.) (CLM) and one-time hand weeding (OHW). The effect of weed management treatment was significant on densities of insect pests, natural enemies and weed and also weed biomass and sesame seed yield. The lowest densities of insect pests including *Myzus persicae*, *Brevicoryne brassicae, Helicoverpa armigera* and *Spodoptera exigua* were observed in CLM treatment. Also, the highest densities of natural enemies *Coccinella septompunctata, Coccinella undecimpunctata* and *Orius niger* were observed in CLM treatment. The highest reductions in grass (51.0%), broadleaf (72.0%), and total (62.6%) weed biomasses and highest seed yield (1456 kg ha^−1^) were obtained in OHW. The seed yields in CLM and WSM treatments were not significantly different with trifluralin treatment and could be recommended in sustainable production of sesame.

Sesame (*Sesamum indicum* L.) is one of the oilseed crops (contains 37–63% of oil), that is cultivated in most of the regions with tropical and subtropical climate in the world^1^. It is adapted to Iran’s climatic conditions^2^ and its growing area in Iran is 60,000 ha with average seed yield of 0.9 t ha^−1^^3^. The sesame yield could be affected by abiotic^4,5^ and biotic stresses such as insect pests^6^ and weeds^7^. In conventional production systems, insecticides and herbicides could be used for pest and weed management of sesame^8^, whereas in sustainable production, using non-chemical management options could improve pest management, maintain seed yield at acceptable level and reduce pesticide application in cropping systems.

Insect pests are the the main factors that reduce the seed yield of sesame^8^. In Iran, sesame leaf roller (*Antigastra catalaunalis*) and foliage feeders of *Neoaliturus haematoceps* and *Empoasca decipiens* cause sever damage^6^. In integrated pest management strategy, using non-chemical methods such as living and straw mulches could be considered. Previuos studies indicated that living mulches could be used for insect pest management^6,9^. Predator populations (*Poecilus chalcites* (Say) and *Scarites quadriceps* Chaudior,) increased in kura clover (*Trifolium ambiguum* M. Bieb.) and alfalfa (*Medicago sativa* L.) living mulches and caused reduction in population of European corn borer (*Ostrinia nubilalis* Hübner)^9^. The population of predators increased in alfalfa living mulch and thereby the density of soybean aphid, *Aphis glycines* Matsumura was decreased^10^. Although, these studies indicate that living mulches could increase predator population, straw mulches also may have similar effects^11^. The higher numbers of carabid beetles, rove beetles and fire ants were captured in sweetpotato (*Ipomoea batatas* (L.) Lam.) plots covered with dead (straw) mulch^12^. The density of lesser cornstalk borer, *Elasmopalpus lignosellus* (Zeller) on bean (*Phaseolus vulgaris* L.), decreased by sunn hemp (*Crotalaria juncea* L.) hay mulch^13^.

The other factor that affect the the seed yield of sesame is weed infestation^8^*.* During the first four weeks of growing season, sesame has low growth rate and also low competitive ability against weeds^8^. The presence of weeds is a major obstacle in sesame production^7,14,15^ and can negatively influence sesame yield. In previous studies the reductions of sesame yield due to uncontrolled weed growth were reported up to 50%^16^ and 74%^17^. Herbicide application has been increased environmental pollution^18^; in addition, herbicide resistance has developed against many weed species. Therefore, using non-chemical weed control options could reduce the production cost and also decrease the negative effects of herbicides on agroecosystems and environment.

In sustainable weed management strategies, using physical, cultural and mechanical controls methods, reduce the herbicide application and improve the crop competitive ability against weeds^19–21^. Straw mulch is the residues from previous crops left on soil surface that prevent light interception by soil surface, reduce seed germination and seedling growth of weeds^22^ and consequently alleviate their competitive ability^23,24^. Living mulch is a cover crop inter-seeded with a main crop and could be used in weed management^25,26^. Living mulches suppress the weeds and improve crop yield, whereas they have lower competitive ability against crops compared to the weeds^24^. Fenugreek (*Trigonella foenum-graecum* L.) and bitter vetch (*Vicia ervilia* (L.) Willd.) have suitable ground cover, improve soil fertility and suppress the weeds^23,27^. Hand weeding is another weed control method that could be used in small farms as a non-chemical and eco-friendly management treatment, although it is laborious and time consuming^28^. In weed management of dill (*Anethum graveolens* L.), the weed control efficacy of one-time hand weeding treatment, was higher than wheat straw mulch, fenugreek and bitter vetch living mulch treatments^29^.

Weeds, insect pests and their natural enemies could be affected by changes in cropping systems^11^. Living and straw mulches may improve weed suppression^30^, provide hiding places to natural enemies and decrease insect pest population^31^. Using non-chemical options would improve the pest and weed management efficiency and maintain the sesame yield at desirable level. Our expectation is that in sesame production, using mulches and hand weeding as sustainable management options, could decrease the insect pest densities and increase the populations of natural enemies and improve the weed control efficacy. So, the purpose of this study was to investigate the effects of living and straw mulches and one-time hand weeding on populations of insect pest and natural enemies, weed suppression and sesame grain yield.

## Results

### Population of insect pests

*Myzus persicae* density was affected significantly by year (*p* ≤ 0.05) and weed management (*p* ≤ 0.05) (Table 1). The *Myzus persicae* density in 2020 was higher than 2021 (Table 2). The highest density of *Myzus persicae* was observed in weed-free treatment and decreased significantly at all treatments and the lowest value was observed in CLM treatment (Table 3). The density of *Myzus persicae* in FLM, VLM and OHW treatments were not significantly different. The *Brevicoryne brassicae* density was affected significantly by year (*p* ≤ 0.05) and weed management (*p* ≤ 0.01) (Table 1). *Brevicoryne brassicae* density in 2021 decreased significantly compared with 2020 (Table 2). The highest and lowest densities of *Brevicoryne brassicae* were observed in weed-free and CLM treatments, respectively (Table 3). The densities of *Brevicoryne brassicae* in trifluralin and weed-infested treatments, were not significantly different. The *Brevicoryne brassicae* density in WSM treatment was higher than FLM, VLM and OHW treatments.

**Table 1:** Analysis of variance for effect of weed management treatments on densities of insect pests and sesame seed yield.

Source of variation	df	Densities of insect pests				Sesame seed yield
		*Myzus persicae*	*Brevicoryne brassicae*	*Helicoverpa armigera*	*Spodoptera exigua*	
Year (Y)	1	*	*	ns	ns	*
Y × Block (Error a)	6	–	–	–	–	–
Management (M)	7	*	**	**	**	**
Y × M	7	ns	ns	ns	ns	ns
Y (Block × M) (Error b)	42	–	–	–	–	–
CV (%)	–	15.32	13.72	6.02	7.65	17.94

ns, * and **: non-significant and significant at *p* ≤ 0.05 and *p* ≤ 0.01, respectively.

**Table 2:** Densities of insect pests and sesame seed yield in 2020 and 2021.

Year	Densities of insect pests				Sesame seed yield (kg ha^−1^)
	*Myzus persicae* (no. twig^−1^)	*Brevicoryne brassicae* (no. twig^−1^)	*Helicoverpa armigera* (no. plant^−1^)	*Spodoptera exigua* (no. plant^−1^)	
2020	188.2 ± 8.5 a	275.2 ± 20.4 a	13.1 ± 3.7 a	16.5 ± 4.7 a	1181 ± 64.3 a
2021	162.1 ± 6.7 b	218.2 ± 17.3 b	11.7 ± 4.5 a	14.7 ± 3.6 a	1002 ± 46.9 b

Values given after means are standard errors (± SE). Means within each column with similar letter are not significantly different at *p* ≤ 0.05 by LSD test.

**Table 3:** Densities of insect pests and sesame seed yield at different weed management treatments.

Weed management treatments	Densities of insect pests				Sesame seed yield (kg ha^−1^)
	*Myzus persicae* (no. twig^−1^)	*Brevicoryne brassicae* (no. twig^−1^)	*Helicoverpa armigera* (no. plant^−1^)	*Spodoptera exigua* (no. plant^−1^)	
Weed-free ^a^	239.1 ± 12.3 a	385.0 ± 16.4 a	19.3 ± 1.1 a	28.9 ± 1.8 a	1670.0 ± 81 a
Trifluralin	149.2 ± 6.3 d	174.8 ± 7.1 e	9.3 ± 0.7 d	11.3 ± 1.5 d	1247.4 ± 75 c
WSM	204.4 ± 8.4 b	307.7 ± 18.3 b	15.9 ± 1.2 b	22.1 ± 2.1 c	1210.5 ± 52 c
FLM	166.3 ± 5.7 c	242. 9 ± 10.5 c	9.7 ± 0.8 d	12.4 ± 1.2 d	927.3 ± 66 d
VLM	175.2 ± 6.1 c	219.6 ± 5.1 d	10.1 ± 0.5 d	13.1 ± 1.6 d	887.2 ± 49 d
CLM	109.4 ± 7.1 e	145.2 ± 8.7 f	5.4 ± 1.4 e	6.2 ± 1.1 f	1155.4 ± 45 c
OHW	175.7 ± 7.2 c	215.7 ± 6.2 d	12.6 ± 0.6 c	17.5 ± 1.4 b	1456.0 ± 56 b
Weed-infested	137.9 ± 9.5 d	171.2 ± 11.3 e	12.8 ± 0.8 c	9.2 ± 0.2 e	536.8 ± 71 e

The presented data are means for 2 years (2020 and 2021). Values given after means are standard errors (± SE). Means within each column with similar letters are not significantly different at *p* ≤ 0.05 by LSD test.

^a^The abbreviations of the weed management treatments were presented in Table 13.

The effect of year was not significant on densities of *Helicoverpa armigera* and *Spodoptera exigua* (Table 1). The effect of weed management treatment was significant (*p* ≤ 0.01) on densities of *Helicoverpa armigera* and *Spodoptera exigua*. The highest densities of these insect pests were observed in weed-free treatment (19.3 and 28.9 no. plant^−1^, respectively) and the lowest values (5.4 and 6.2 no. plant^−1^, respectively) in CLM treatment (Table 3). There were no significant differences in densities of *Helicoverpa armigera* and *Spodoptera exigua* in VLM, FLM and trifluralin treatments. The densities of all insect pests were not significantly affected by interaction effect of year × weed management (Table 1).

### Population of natural enemies

The effects of year and interaction effect of year × weed management treatment were not significant on densities of all natural enemies (Table 4). The effect of weed management treatment was significant (*p* ≤ 0.01) on natural enemies densities (Table 4). The highest densities of *Coccinella septompunctata, Coccinella undecimpunctata* and *Orius niger* were obtained in CLM treatment (14.4, 11.3 and 21.1 no. plant^−1^, respectively) and the lowest values (3.3, 2.1 and 5.6 no. plant^−1^, respectively) in weed-free treatment (Table 5). The densities of *Coccinella septompunctata* were not significantly different in trifluralin, FLM and VLM treatments. The densities of *Coccinella septompunctata* and *Orius niger* were not significantly different in FLM, VLM and OHW treatments (Table 5).

**Table 4:** Analysis of variance for effect of weed management treatments on densities of natural enemies.

Source of variation	df	Densities of natural enemies		
		*Coccinella septompunctata*	*Coccinella undecimpunctata*	*Orius niger*
Year (Y)	1	ns	ns	ns
Y × Block (Error a)	6	–	–	–
Management (M)	7	**	**	**
Y × M	7	ns	ns	ns
Y (Block × M) (Error b)	42	–	–	–
CV (%)	–	6.02	7.65	7.32

ns, * and **: non-significant and significant at *p* ≤ 0.05 and *p* ≤ 0.01, respectively.

**Table 5:** Densities of natural enemies in different weed management treatments.

Weed management treatments	Densities of natural enemies		
	*Coccinella septompunctata* (no. m^−2^)	*Coccinella undecimpunctata* (no. m^−2^)	*Orius niger* (no. m^−2^)
Weed-free^a^	3.3 ± 0.4 e	2.1 ± 0.3 e	5.6 ± 0.8 d
Trifluralin	10.6 ± 0.9 b	6.3 ± 0.2 c	9.2 ± 1.2 c
WSM	5.3 ± 0.6 d	3.9 ± 0.2 d	8.4 ± 1.3 c
FLM	11.1 ± 0.8 b	8.4 ± 0.8 b	14.9 ± 1.4 b
VLM	10.5 ± 1.0 b	8.9 ± 0.9 b	16.9 ± 1.1 b
CLM	14.4 ± 1.4 a	11.3 ± 1.1 a	21.1 ± 2.1 a
OHW	8.1 ± 0.7 c	7.9 ± 0.7 b	15.6 ± 1.3 b
Weed-infested	8.3 ± 0.8 c	6.2 ± 0.3 c	8.9 ± 1.4 c

The presented data are means for 2 years (2020 and 2021). Values given after means are standard errors (± SE). Means within each column with similar letters are not significantly different at *p* ≤ 0.05 by LSD test.

^a^The abbreviations of the weed management treatments were presented in Table 13.

### Weed density

The weed species composition in experimental field was presented in Table 6. The grass, broadleaf and total weed densities were not affected significantly by year and interaction effect of year × management treatment (Table 7), but affected significantly (*p* ≤ 0.01, *p* ≤ 0.05 and *p* ≤ 0.05, respectively) by weed management treatment. In weed-infested treatment, the grass weed density was higher than that of broadleaf weed. The grass and broadleaf weed densities decreased significantly at all weed management treatments compared with weed-infested treatment and the highest reductions (76.2 and 89.7%, respectively) were observed in OHW treatment (Table 8). At all living mulch treatments (FLM. VLM and CLM), the grass weed densities were higher than WSM treatment. The broadleaf weed densities in FLM and VLM treatments were higher than CLM and WSM treatments. The total weed density decreased significantly at all weed management treatments compared with weed-infested treatment and the highest reductuion (82.4%) was observed in OHW treatment (Table 8). In CLM, VLM and FLM treatments, the weed densities were not significantly different. The grass, broadleaf and total weed densities in trifluralin treatments were lower than straw mulch and all living mulch treatments (FLM. VLM and CLM).

**Table 6:** Common name, scientific name, family name and morphology of identified weed species in experimental field.

No	Common name	Scientific name	Family name	Morphology
1	Yellow nutsedge	*Cyperus esculanthus* L.	Cyperaceae	Grass
2	Johnsongrass	*Sorghum halepens* L.	Poaceae	Grass
3	Green foxtail	*Setaria viridis* L.	Poaceae	Grass
4	Common purslane	*Portulaca oleracea* L.	Portulacaceae	Broadleaf
5	Wild radish	*Raphanus raphanistrum* L.	Brassicaceae	Broadleaf
6	Field bindweed	*Convolvulus arvensis* L.	Convolvulaceae	Broadleaf
7	Milk thistle	*Silybum marianum* L.	Asteraceae	Broadleaf
8	Bermuda grass	*Cynodon dactylon* L.	Poaceae	Grass
9	Black nightshade	*Solanum nigrum* L.	Solanaceae	Broadleaf

**Table 7:** Analysis of variance for effect of weed management treatments on grass, broadleaf and total weed density.

Source of variation		Grass weed density	Broadleaf weed density	Total weed density
Year (Y)	1	ns	ns	ns
Y × Block (Error a)	6	–	–	–
Management (M)	6	**	*	*
Y × M	6	ns	ns	ns
Y (Block × M) (Error b)	36	–	–	–
CV (%)	–	11.15	14.73	13.25

ns, * and **: non-significant and significant at *p* ≤ 0.05 and *p* ≤ 0.01, respectively.

**Table 8:** Effect of different weed management treatments on grass, broadleaf and total weed density.

Weed management treatments	Grass weed density (Plant m^−2^)	Broadleaf weed density (Plant m^−2^)	Total weed density (Plant m^−2^)
Trifluralin^a^	27.6 ± 4.3 d	14.7 ± 2.3 e	42.3 ± 4.3 d
WSM	34.3 ± 2.1 c	22.9 ± 2.5 d	57.2 ± 4.7 c
FLM	39.3 ± 2.2 b	38.7 ± 4.6 b	78.0 ± 5.2 b
VLM	44.0 ± 6.9 b	35.3 ± 2.4 b	79.3 ± 7.5 b
CLM	42.0 ± 5.8 b	30.3 ± 1.4 c	72.3 ± 6.1 b
OHW	17.4 ± 7.5 e	6.3 ± 7.5 f	23.7 ± 7.5 e
Weed-infested	73.0 ± 9.3 a	61.3 ± 8.3 a	134.3 ± 12.6 a

The presented data are means for 2 years (2020 and 2021). Values given after means are standard errors (± SE). Means within each column with similar letters are not significantly different at *p* ≤ 0.05 by LSD test.

^a^The abbreviations of the weed management treatments were presented in Table 13.

### Weed biomass

The effects of year and interaction effect of year × management treatment were not significant on grass, broadleaf and total weed biomasses (Table 9). The effect of weed management treatment was significant on grass (*p* ≤ 0.01), broadleaf (*p* ≤ 0.01) and total weed (*p* ≤ 0.01) biomasses. In weed-infested treatment, the broadleaf weed biomass was higher than that of grass weed. At all weed management treatments the grass and broadleaf weed biomasses decreased significantly compared with weed-infested treatment (Table 10). The highest reductions in grass and broadleaf weed biomasses (51.0 and 72.0%, respectively, compared with weed-infested) were observed in OHW treatment. At WSM and all living mulch treatments (FLM, VLM and CLM), the grass weed biomasses were higher than trifluralin treatment. The broadleaf weed biomasses at all living mulch treatments were higher than WSM and trifluralin treatments. The total weed biomass decreased significantly at all weed management treatments compared with weed-infested treatment and the highest reductuion (62.6%) was observed in OHW treatment (Table 10). In CLM, VLM and FLM treatments, the total weed biomasses were not significantly different. The total weed biomasses in trifluralin and WSM treatments were lower than all living mulch treatments (FLM, VLM and CLM).

**Table 9:** Analysis of variance for effect of weed management treatments on grass, broadleaf and total weed biomass.

Source of variation		Grass weed biomass	Broadleaf weed biomass	Total weed biomass
Year (Y)	1	ns	ns	ns
Y × Block (Error a)	6	–	–	–
Management (M)	6	**	**	**
Y × M	6	ns	ns	ns
Y (Block × M) (Error b)	36	–	–	–
CV (%)	–	11.15	14.73	11.72

ns, * and **: non-significant and significant at *p* ≤ 0.05 and *p* ≤ 0.01, respectively.

**Table 10:** Effect of different weed management treatments on grass, broadleaf and total weed biomass.

Weed management treatments	Grass weed biomass (Plant m^−2^)	Broadleaf weed biomass (Plant m^−2^)	Total weed biomass (Plant m^−2^)
Trifluralin^a^	70.4 ± 4.3 c	60.6 ± 6.2 c	131.0 ± 9.1 d
WSM	89.5 ± 4.7 b	64.3 ± 6.5 c	153.8 ± 8.8 c
FLM	93.0 ± 5.2 b	90.6 ± 7.2 b	183.6 ± 11.3 b
VLM	101.5 ± 7.5 b	95.3 ± 6.7 b	196.8 ± 15.2 b
CLM	96.1 ± 6.1 b	83.6 ± 7.4 b	179.7 ± 11.3 b
OHW	61.1 ± 3.2 d	43.2 ± 5.1 d	104.3 ± 8.9 e
Weed-infested	124.6 ± 9.3 a	154.3 ± 13.1 a	278.9 ± 24.2 a

The presented data are means for 2 years (2020 and 2021). Values given after means are standard errors (± SE). Means within each column with similar letters are not significantly different at *p* ≤ 0.05 by LSD test.

^a^The abbreviations of the weed management treatments were presented in Table 13.

### Sesame seed yield

The effects of year (*p* ≤ 0.05) and weed management treatment (*p* ≤ 0.01) were significant on sesame seed yield (Table 1). The sesame seed yield in 2021 (1002 kg ha^−1^) decreased significantly compared with 2020 (1181 kg ha^−1^) (Table 2). The weed-free treatment had the greatest sesame seed yield (1670 kg ha^−1^) and in other treatments, the seed yield decreasd significantly (Table 3). The OHW treatment had the greatest seed yield (1456 kg ha^−1^) among the weed management treatments. There was no significant difference among the seed yields in trifluralin, CLM and WSM treatments. Also, among the living mulch treatments, the seed yields in VLM and FLM treatments, decreased compared with CLM (Table 3). The interaction of year × weed management treatment was not significant on seed yield.

## Discussion

The densities of *Myzus persicae* and *Brevicoryne brassicae* in 2020 were higher than 2021. This increase could be attributed to higher mean temperatures in 2020 (from April to August; Table 1) compared to 2021. El Fakhouri et al.^32^, also observed that increasing the temperature promoted pea aphid (*Acyrthosiphon pisum* Harris) population in lentil (*Lens culinaris* Medikus). Also, the precipitation in 2020 growing season (from May to September) was higher than 2021, that could be another reason for increasing the densities of *Myzus persicae* and *Brevicoryne brassicae*. Frank and Liburd^33^ also reported that the differences in temperature and rainfall could affect the population densities of aphids. The highest densities of all insect pests were observed in weed-free treatment and reduced significantly in weed-infested and all weed management treatments. These results indicate that the presence of weeds or living mulches in sesame field would reduce the densities of these insect pests. The densities of *Myzus persicae* and *Brevicoryne brassicae* in WSM treatment were higher than those in VLM and FLM treatments, that are in consistent with findings of Frank and Liburd^33^. They found that in Zucchini, (*Cucurbita pepo* L.), the aphid density in synthetic white mulch treatment increased compared with living mulches of white clover (*Trifolium repens* L.) and buckwheat (*Fagopyrum esculentum* Moench). Conversely, in bush bean (*Phaseolus vulgaris*), Gill et al.^11^ observed that aphid densities in living and straw mulches and unmulched control, were not significantly different. The lowest densities of *Myzus persicae* and *Brevicoryne brassicae* were observed in CLM treatment that is in agreement with Zhao et al.^34^ which reported that the presence of *Calendula officinalis* enhanced *Myzus persicae* suppression. Also, in intercropping of cowpea (*Vigna unguiculata* L. Walp.) and African marigold (*Tagetes erecta* L.) with *Cucurbita pepo*, observed that marigold and marigold-cowpea intercropping suppressed the aphids by increase in population of natural enemies^35^.

The lowest densities of *Spodoptera exigua* and *Helicoverpa armigera* were also observed in CLM treatment, as Fabrick et al.^36^ reported that survival of *Lygus hesperus* and *Bemisia tabaci* reduced on French marigold (*Tagetes patula* L.) plants compared with common bean (*Phaseolus vulgaris*). However, the biotic mechanisms that confer such repellency are often not well understood^37^ and needs more investigation. Moreover, the reductions in insect pests densities in this treatment (CLM) could be related to increase in densities of natural enemies. In WSM treatment, the densities of these insect pests were higher than VLM and FLM living mulch treatments. Frank and Liburd^33^ also reported that the densities of whitefly (*Bemisia argentifolii* Bellows & Perring) in synthetic white and reflective mulches were higher than those of *Fagopyrum esculentum* and *Trifolium repens* living mulches. Bruce et al.^30^ also observed that in *Cucurbita pepo* production, the cucumber beetles (*Acalymma trivittatum*) density in straw mulch treatment, increased compared with those in annual ryegrass (*Lolium multiflorum*) and white clover (*Trifolium repens*) living mulches.

The highest densities of all natural enemies (*Coccinella septompunctata*, *Coccinella undecimpunctata* and *Orius niger*) were observed in CLM treatment that caused significant reduction in all insect pest densities. Similar results have been reported in previous studies on pot marigold *Calendula officinalis*^34^ and African marigold *Tagetes erecta* L.^35,38^. The presence of *Calendula officinalis* flowers might attract natural enemies into crop fields without any direct effects on natural enemy fitness and improve attack rates on the pest^39^, or it might increase longevity or fecundity of natural enemy^40,41^. Zhao et al.^34^ reported that the growth rate and number of *Orius sauteri*, increased significantly in presence of *Calendula officinalis*, that consequently increased the *Myzus persicae* suppression.

All living mulch treatments (FLM, VLM and CLM) had higher densities of natural enemies than WSM treatment. Also, the densities of natural enemies in weed-infested treatment were higher than weed-free treatment. The hypothesis of natural enemy was confirmed by these results, where the increase in densities of natural enemies is the outcome of the increased plant diversity^42^. Frank and Liburd^33^ also reproted that the populations of natural enemies in living mulches were higher than control (bare ground) and synthetic mulches. Interplanting of red clover (*Trifolium pratense* L.) with cucumber (*Cucumis sativus* L.), increased the densities of natural enemies and decreased the densities of melon aphid (*Aphis gossypii*) and cucumber beetles (*Acalymma trivittatum*)^43^. Also, the living mulch of buckwheat (*Fagopyrum esculentum*) increased the population of natural predator^44^.

At all weed management treatments except FLM, the reductions in grass weed densities compared with weed-infested treatment, were lower than those in broadleaf weed densities. The greatest reductions in grass, broadleaf and total weed densities were observed in OHW treatment that is in agreement with previous studies on *Anethum graveolens* L.^29^ and *Glycine max* L.^45^. In WSM treatment, the reductions in grass, broadleaf and total weed densities were greater than VLM and FLM treatments that may be attributed to the higher ground cover in this treatment than living mulch treatments (VLM and FLM)^46^. Straw mulches could suppress seedling emergence of weeds through physical barrier created by mulch itself^47^ and reduction in light interception by seeds^48,49^. In trifluralin treatment, the reductions in grass, broadleaf and total weed densities were greater than straw (WSM) and living mulches (FLM, VLM and CLM) that could be explained by inhibition effect of trifluralin (as a soil-applied herbicide) on weed seed germination^28^.

At all weed management treatments, the reductions in grass weed biomasses compared with weed-infested treatment were lower than those in broadleaf weed biomasses. The greatest reductions in grass, broadleaf and total weed biomasses were observed in OHW treatment. The *Calendula officinalis* living mulch (CLM) and wheat straw mulch (WSM) indicated higher weed biomass reduction (weed control efficacy) than *Trigonella foenum-graecum* (FLM) and *Vicia ervilia* (VLM) living mulches. In *Anethum graveolens*, the weed biomass reductions in *Trigonella foenum-graecum* and *Vicia ervilia* living mulches were lower than wheat straw mulch^29^. For weed supression, living mulches should have high initial growth rate and competitive ability against weeds^50^. The weed biomass reduction in trifluralin treatment was greater than straw and living much treatments (WSM, FLM, VLM and CLM) that are in agreement with previous findings on *Dracocephalum moldavica*^28^, *Anethum graveolens*^29^ and *Cuminum cyminum*^51^.

Among the weed management treatments, the greatest sesame seed yield was obtained in OHW treatment that could be due to the greatest weed biomass reduction (62.6%) in this treatment. Similar results have been reported in previuos studies on *Phaseolus vulgaris* L.^52^ and *Anethum graveolens* L.^29^, whereas they observed that the grain yields in herbicide application, living mulch and straw mulch treatments, were lower than one-time hand weeding treatment. The highest reductions in seed yield were observed in FLM (44.5% comared to weed-free) and VLM (46.9% comared to weed-free) treatments that may be related to the competition occurred by living mulch treatment as reported in previous studies^29,50^. The sesame seed yield in CLM treatment, enhanced compared with VLM and FLM treatments, while the weed biomasses at all living mulches were not significantly different. These results could be explained by increasing the densities of natural enemies and suppression of insect pests in CLM treatment. The sesame seed yields were not significantly different in WSM and trifluralin application, but the weed biomass reduction in trifluralin treatment (53.1%) was higher than WSM treatment (44.9%). Also the densities of all insect pests in trifluralin treatment were lower than WSM treatment. Therefore, the reason for non-significant difference between sesame seed yields in trifluralin and WSM treatments may be related to increase in water content of soil due to straw mulch coverage^29,53^.

## Conclusions

Total weed densities and biomasses were not significantly different in CLM, VLM and FLM treatments (living mulches), while the sesame seed yield in CLM treatment was higher than FLM and VLM treatments. The increase in seed yield in CLM treatment compared with other living mulches, could be explained by increase in densities of natural enemies and thereby reduction in densities of all insect pests in this treatment. The densities of all natural enemies in OHW treatment were higher than WSM treatment, which caused reduction in all insect pest densities in this treatment. The highest reduction in total weed biomass and the greatest seed yield were obtained in OHW treatment, threrfore in cropping systems with no labour limitation, the growers could use this method for weed management. The sesame seed yield in trifluralin treatment was not significantly different with CLM and WSM treatments and in sustainable production systems, these non-chemical treatments could be recommended instead of trifluralin for weed management in sesame. One-time hand weeding (OHW) was more efficient for weed suppression than straw and living mulches. This result indicates that developing other weed management methods such as machine powered hoeing and harrowing could improve the weed and pest management, thereby the seed yield in sesame. Also, more investigations are required to investigate the effects of sowing time of living mulch relative to main crop and other types of straw and living mulches in sustainable weed and pest management of sesame.

## Materials and methods

### Experimental site

A field study was carried out at East Azarbayjan, Iran (Latitude 38° 53′ N, Longitude 46° 47′ E, Altitude 315 m a.s.l.) during 2020–2021. The experimental area is warm temperate with mean annual precipitation of 375 mm mean annual temperature of 25.8 °C. The 2-year (2020 and 2021) data of monthly total precipitation and mean temperature of the experimental site are presented in Table 11. The properties of soil of experimental field at depth of 0–30 cm are presented in Table 12.

**Table 11:** Mean temperature (°C) and precipitation (mm) in Khoda-Afarin, East Azarbaijan, Iran during the sesame growing seasons in 2020 and 2021.

Month	2020		2021	
	Mean temperature	Precipitation	Mean temperature	Precipitation
April	17.6	86.3	12.9	45.9
May	24.1	106.8	21.6	82.4
June	28.8	46.7	26.8	22.6
July	35.6	21.2	33.4	14.7
August	39.7	12.6	38.2	11.2
September	31.7	68.6	32.1	14.5
October	24.9	32.4	26.3	8.8

**Table 12:** Physico-chemical properties of the soil of experimental area in depth of 0–30 cm.

Parameter	Value
Soil texture	Loam
Clay (%)	14
Silt (%)	38
Sand (%)	48
pH	7.91
OC (%)	2.66
EC (dS m^−1^)	1.48
N (%)	0.26
P (mg kg^−1^)	41.4
K (mg kg^−1^)	76.5

### Field practice and experimental procedure

The experiments were arranged as RCBD (randomized complete block design) with eight treatments and four replicates. The weed management treatments were including application of trifluralin (960 g ai ha^−1^ as pre-planting of sesame), using straw mulch of wheat (5.0 t ha^−1^) (WSM), living mulches of fenugreek (*Trigonella foenum-graecum* L.) (FLM), bitter vetch (*Vicia ervilia* L.) (VLM) and calendula (*Calendula officinalis* L.) (CLM) and one-time hand weeding (OHW) at 50 days after planting (DAP) of sesame. The weed-infested and weed-free treatments during whole growing season also were considered in the experiment (Table 13). In weed-free treatments the weeds were removed manually every day. 12 planting rows spaced 50 cm apart with 5 m length (6 m × 5 m) was considered as an experimental plot.

**Table 13:** Details of weed management treatments used in sesame.

Weed management treatments	Abbreviation
Weed-free during whole season	Weed-free
Recommended dose of trifluralin (960 g ai ha^−1^) pre-planting of sesame with soil incorporation	Trifluralin
Wheat straw mulch (5.0 t ha^−1^)	WSM
Fenugreek (*Trigonella foenum-graecum* L.) living mulch	FLM
Bitter vetch (*Vicia ervilia* L.) living mulch	VLM
Calendula (*Calendula officinalis* L.) living mulch	CLM
One-time hand weeding treatment (50 days after sesame planting)	OHW
Weed-infested during whole season	Weed-infested

Deep moldboard plowing (25–30 cm) in the spring which was followed by disking before planting was used for seedbed preparation. The soil-applied herbicide, trifluralin (Treflan, EC, 480 g L^−1^, Aria Shimi) was applied as pre-planting in to the top soil layer (5-cm); the soil incorporation was done by raking instantly after herbicide use. For trifluralin application a backpack sprayer (Matabi, Goizper Group, Spain) was used equipped with Flood-jet nozzle which calibrated to deliver 265 L ha^−1^ at 235 kPa. The sowing of sesame seeds was done by hand on 15 May 2020 and 17 May 2021 in soil depth of 2 cm with 18.18 plants m^−2^ density. The planting row distance was 50 cm and the plant distance over the row was 11 cm. The straw mulch of wheat (5.0 t ha^−1^) was applied between the sesame rows, immediately after planting. The *Calendula officinalis, Trigonella foenum-graecum* and *Vicia ervilia* seeds were planted (simultaneously with crop) as living mulches between the sesame rows, at seeding rates of 15, 35 and 40 kg ha^−1^, respectively. The growing period of *Calendula officinalis, Trigonella foenum-graecum*, *Vicia ervilia* and were 108, 117 and 75 days, respectively. The one-time hand weeding treatment was done 50 DAP of sesame. After planting of sesame and living mulches, the furrow irrigation was done in experimental plots and it was done every 7-day until physiological maturity of sesame.

### Ethical approval

This experimental research upon plants complies with relevant institutional, national, and international guidelines and legislation. The seeds of sesame, fenugreek, bitter vetch and calendula were purchased from Tabriz, East Azarbayjan, Iran.

### Populations of pests and natural enemies

At 7-day intervals, the densities of pests and natural enemies at different treatments were recorded from their appearance time in flowering stage of sesame (52 DAP; 05 July 2020 and 07 July 2021) until sesame physiological maturity (12 September 2020 and 16 September 2021). To determine the population of aphids in each plot, *Myzus persicae* and *Brevicoryne brassicae* were randomly sampled in 20 10-cm twigs in the morning (8:30–11:00 a.m)^32^. For *Helicoverpa armigera* and *Spodoptera exigua* at each sampling time, 10 plants were randomly selected in experimental plots and the number of larvae on each plant was counted.

To evaluate the population of natural enemies, *Coccinella septompunctata*, *Coccinella undecimpunctata* and *Orius niger*; the sampling was done every week in 5 × 5 m experimental plots. *Coccinella septompunctata* and *Coccinella undecimpunctata* were sampled using a 1 × 1 m quadrat. At each plot, two quadrats were thrown randomly and the ladybird beetles of both species were counted. For data analysis, the mean number of ladybird beetles counted in the two quadrats, were used. A sweep net (38 cm diameter ring and 80 cm light wooden handle) was used for sampling of *Orius niger*. For a sampling unit, ten 180◦ sweeps per plot was considered. The insect pests and natural enemies were stored in 70% ethanol in lab condition in vials.

### Weed traits

At maturity stage of sesame (121 DAP in 2020 and 123 DAP in 2021) in order to measure grass, broadleaf and total weed density and biomass the weeds were cutted from 1.5 m^−2^ area (using three 1 m × 0.5 m quadrats). The weed species in the experimental field were included *Cyperus esculanthus* L., *Sorghum halepens* L*.*, *Setaria viridis* L., *Portulaca oleracea* L*.*, *Raphanus raphanistrum* L*.*, *Convolvulus arvensis* L*.*, *Silybum marianum* L*.*, *Cynodon dactylon* L., and *Solanum nigrum* L. The grass, broadleaf and total weed densities and biomasses were measured separately. For weed biomass measurement, the weeds were placed in paper bags; oven-dried at 75 °C for 48 h, and weighed.

### Sesame seed yield

To determine the seed yield, an area of 2 m^2^ was harvested from the middle rows of all experimental plots at maturity stage, on 12 September 2020 and 16 September 2021. The mechanical thresher was used to obtain the seed yield (kg ha^−1^) from harvested plants in each plot.

### Statistical analysis

A combined analysis of variance (ANOVA) was performed using SPSS software ver.20 based on RCBD with four replications. The data for densities of pests and natural enemies, weed density and biomass and sesame seed yield, met the assumptions of normality and homogeneity of variance and no transformation was needed. For means comparison, the Fisher’s Protected Least Significance Difference test (*p* ≤ 0.05) was used.

## Data Availability

The necessary information is available from the corresponding author on reasonable request.
